# Disentangling Genetic and Environmental Effects on the Proteotypes of Individuals

**DOI:** 10.1016/j.cell.2019.03.015

**Published:** 2019-05-16

**Authors:** Natalie Romanov, Michael Kuhn, Ruedi Aebersold, Alessandro Ori, Martin Beck, Peer Bork

**Affiliations:** 1 Structural and Computational Biology Unit, European Molecular Biology Laboratory, Heidelberg, Germany; 2 Department of Biology, Institute of Molecular Systems Biology, ETH Zurich, Switzerland; 3 Faculty of Science, University of Zurich, Zurich, Switzerland; 4 Leibniz Institute on Aging – Fritz Lipmann Institute, Jena, Germany; 5 Cell Biology and Biophysics Unit, European Molecular Biology Laboratory, Heidelberg, Germany; 6 Max Delbrück Center for Molecular Medicine, Berlin, Germany

## Abstract

Proteotypes, like genotypes, have been found to vary between individuals in several studies, but consistent molecular functional traits across studies remain to be quantified. In a meta-analysis of 11 proteomics datasets from humans and mice, we use co-variation of proteins in known functional modules across datasets and individuals to obtain a consensus landscape of proteotype variation. We find that individuals differ considerably in both protein complex abundances and stoichiometry. We disentangle genetic and environmental factors impacting these metrics, with genetic sex and specific diets together explaining 13.5% and 11.6% of the observed variation of complex abundance and stoichiometry, respectively. Sex-specific differences, for example, include various proteins and complexes, where the respective genes are not located on sex-specific chromosomes. Diet-specific differences, added to the individual genetic backgrounds, might become a starting point for personalized proteotype modulation toward desired features.

## Highlights

• Benchmarking of datasets on human and mouse proteotypes
• Consistent co-variation landscape of functional modules across individuals
• Protein complexes vary in their stoichiometry across individuals
• Quantifying effects of genetic sex and specific diets on complexes

## Graphical Abstract

**Figure:**
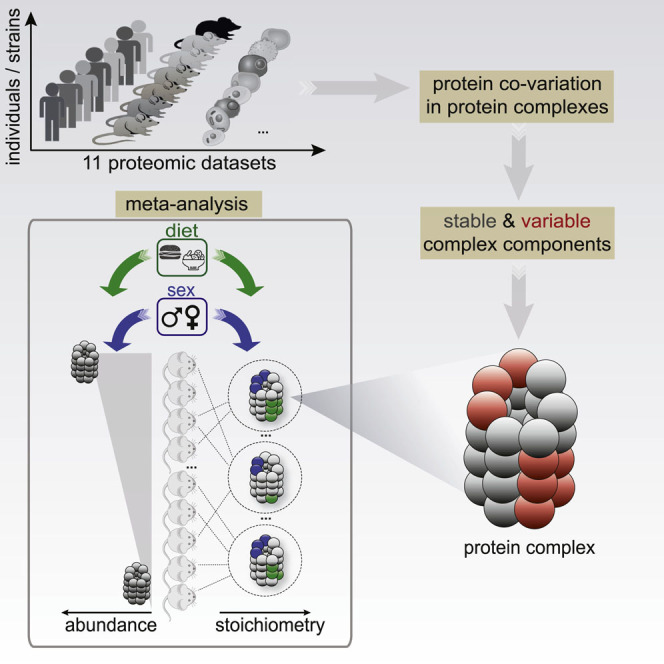

## Teaser

Protein functional module variation between individuals might serve as molecular fingerprints for a wide range of environmental and genetic factors that are tunable by intervention toward desired proteotypes.

## Introduction

Recent advances in the experimental throughput of mass spectrometry (MS)-based proteomics have enabled large-scale studies of proteotypes, defined as the proteome complement of a genotype (Picotti et al., 2013), which can be obtained for cell lines or tissues. Although genotype and proteotype are poorly correlated (Liu et al., 2016), genetic variation has been shown to have a considerable impact on the abundance of proteins across yeast strains (Picotti et al., 2013), mouse strains (Wu et al., 2014, Williams et al., 2016, Chick et al., 2016), fly strains (Okada et al., 2016), and human individuals (Battle et al., 2015, Wu et al., 2013, Liu et al., 2015). While some rare diseases are 100% genetically determined, for most common ones, the genetic contribution is minor and environmental factors play an important role. In obesity, for example, only ∼6% of the phenotypic variance can be explained by the associated genetic variance (Speliotes et al., 2010). The identification of functional traits in proteotypes therefore holds great promise to provide disease-associated fingerprints in individuals because such traits should be a molecular reflection of not only genetic but also environmental factors (e.g., life style). If environmental factors can be disentangled from genetic ones, such fingerprints might even provide a basis for personalized treatments.

Establishing such connections from genetic or environmental factors to the individual proteotype remains challenging, however. This is due to technical limitations, in particular the variable experimental noise across studies, but also biological buffering mechanisms (Stefely et al., 2016). However, the modular architecture of the proteome (i.e., its organization into complexes, pathways, and subcellular structures) provides powerful means to overcome these issues by interpreting observed variations in the context of well-established biological functions (Stefely et al., 2016, Ori et al., 2016, Parca et al., 2018).

Several seminal studies have shown the variability of protein abundances across individuals in human and mice (Wu et al., 2013, Wu et al., 2014, Chick et al., 2016, Battle et al., 2015, Liu et al., 2015, Gonçalves et al., 2017). Although each study highlighted individual proteins or functional modules that were found to be variable, a systematic and unbiased analysis of functional modules across multiple studies is lacking. It remains unknown if alterations of specific cellular functions are prevalent and at which organizational level such alterations manifest (e.g., complexes, pathways, organelles). Furthermore, the extent to which the proteome of individuals is variable, and how this variability is linked to environmental or genetic factors remains difficult to estimate.

A case in point is the lack of stratification of male and female organisms at the proteotype level. Various studies have reported protein abundance variation due to the genetic sex of an organism, but focus only on chromosome X/Y-specific protein expression rather than on the systemic differences in the overall proteotypic patterns (Wu et al., 2013, Kukurba et al., 2016). Exploring gender differences of the proteome is pivotal for our understanding of clinical phenotypes that are often sexually dimorphic (Naugler et al., 2007).

Here, we analyze 11 public datasets to investigate proteotypes of healthy and diseased individuals from human and mice. The proteotype of an individual describes, more generally, the state of a proteome (i.e., protein abundances, connectivity, turnover, and localization) in conjunction with the presence and state of posttranslational modifications. It differs from cell type to cell type (Geiger et al., 2012, Uhlén et al., 2015, Ori et al., 2016) and changes over time (Ori et al., 2015, Cellerino and Ori, 2017). The 11 datasets, however, only describe protein abundances and source information (a given cell line or tissue). Therefore, we use an operational definition of the proteotype that is restricted to protein abundances. For each dataset, we test to what extent it recovers known functional modules and assess the contribution of these modules to proteotype variation and their association with genetic and environmental factors. Our unbiased analysis reveals that protein complex abundance and stoichiometry are the major determinants of an individual’s proteome, while proteins in other functional modules, such as molecular pathways, co-vary less often across individuals. Within protein complexes, the consistently co-varying dynamic components can be associated with both genetic and environmental factors. We demonstrate that sex as a genetic factor explains the largest fraction of the observed variability in protein complexes but also find functional modules that are impacted by diet, an environmental factor. As both examples alone already have considerable effect sizes, our study implies that protein functional module variation might serve as a molecular fingerprint of a wide range of environmental and genetic factors, which might be tunable toward desired proteotypes (e.g., by individualized diets) (Zeevi et al., 2015).

## Results

### Interacting Proteins Co-vary across Healthy Individuals

**Figure 1:**
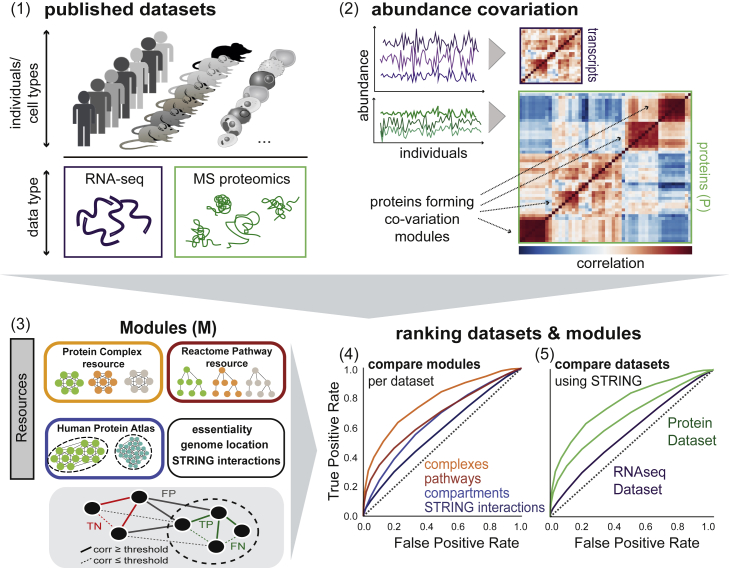
Schematic Illustration of Workflow (1) Published proteomics datasets on human individuals, mouse strains, and cell types are considered for the study (Table S1). If available, RNA-seq datasets for the respective specimens are also taken into account. (2) Co-variation of protein (or transcript) abundances is calculated for each dataset. (3) We integrate resources on protein modules (STRING protein interactions, protein complexes, Reactome pathways, Human Protein Atlas cellular localization, etc.) to reveal co-varying modules across individuals. The schematic below illustrates the definition of true positive (TP), false positive (FP), false negative (FN), and true negative (TN) interactions based on whether the interaction occurs within a module (dashed circle) or outside at a given correlation (corr) threshold. Iterating through correlation thresholds gives the receiver operating characteristics (ROC). (4) Different modules are then compared by the ROC metrics in each dataset (recovery of known modules). (5) Datasets can be compared by the degree of recoverable known co-variation (STRING interactions). See also Figure S1 and Table S1.

Proteins are not functioning alone, but are organized into functional modules and networks, spanning from complexes to pathways and entire organelles. In order to understand which features or levels of organization define the proteome state of individuals, we tested to what extent known functional modules or protein associations can be recovered in different proteomics datasets. Implicitly, we thereby tested the power of each dataset and ensured consistent results. We examined datasets resulting from profiling proteins across cancer patients (The Cancer Genome Atlas [TCGA] panels: Ovarian Cancer, Zhang et al., 2016; Breast Cancer, Mertins et al., 2016; and Colorectal Cancer, Zhang et al., 2014), healthy human individuals (Battle et al., 2015, Wu et al., 2013, Khan et al., 2013), and healthy mouse strains that were exposed to different diets (Wu et al., 2014, Chick et al., 2016) and compared them to other proteomic datasets derived from cell types (Geiger et al., 2012) (Figure 1; Table S1). The respective studies differed with respect to the MS-technique employed for protein measurement, as well as the source organism, resolution (tissues or specific cells), and organ or cell type (Table S1). While Wu et al. (2014), Williams et al. (2016); BXD80 mouse strains, and Chick et al. (2016) (diversity outbred [DO] mouse strains and Founder mouse strains) recovered proteins from mouse livers from different mouse populations, Battle et al. (2015) extracted proteins from lymphoblastoid cell lines (LCLs) of human individuals (HapMap Yoruba individuals). If available, we included transcriptional data as well as data derived from ribosome profiling to reveal the impact of transcriptional and translational regulation.

**Figure S1:**
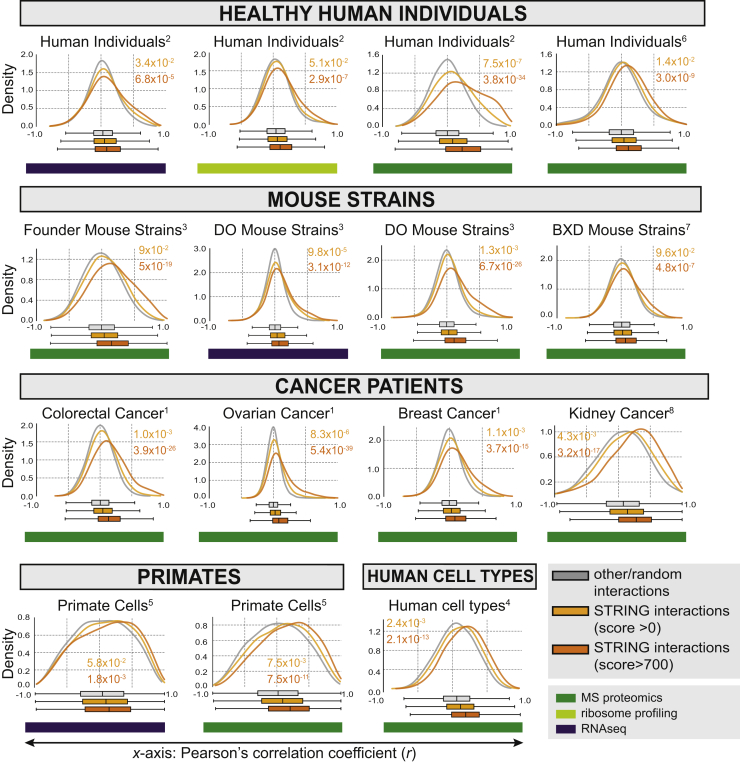
Recovery of Known STRING Interactions in Different Published Datasets, Related to Figure 1 For all datasets considered for the ROC-analysis, the distribution of Pearson correlation coefficients for protein-protein pairs with STRING interaction score > 700 (combined score, orange), known interaction (combined score > 0, yellow) and random protein-protein pairs (gray) is shown. The Mann-Whitney *U*-test was used to assess significance of the respective shifts (indicated in colored *p-value*s). The number next to the dataset name indicates the reference for the respective dataset, with (1) referring to the The Cancer Genome Atlas (TCGA) publications, (2) Battle et al. (2015), (3) Chick et al. (2016), (4) Geiger et al. (2012), (5) Khan et al. (2013), (6) Wu et al. (2013), (7) Williams et al. (2016), and (8) Guo et al. (2015) (same numbering as for Figure 2). More details on the datasets are given in Table S1.

**Figure 2:**
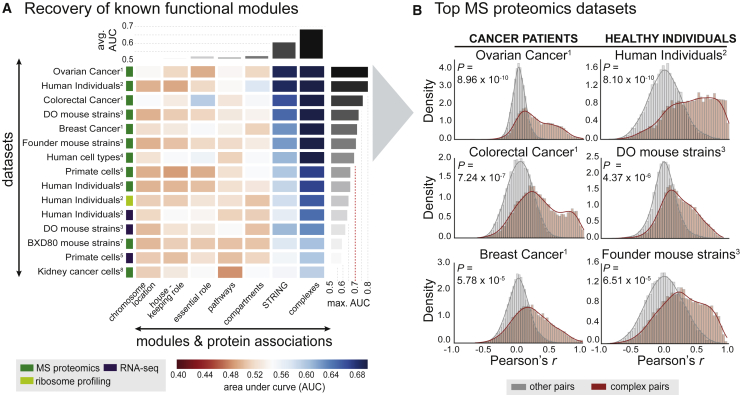
The Strongest Co-variation across Individuals Stem from Protein Complexes (A) Recovery of known functional modules by means of receiver operating characteristic (ROC)-analysis. Each cell of the matrix displays the AUC (area under curve) value for a given module (x axis) in the given dataset (y axis). Modules or protein associations are ordered according to respective average AUC. The type of data is indicated by the colored boxes next to the dataset. The number indicates the reference for the respective dataset, with (1) referring to the The Cancer Genome Atlas (TCGA) publications, (2) Battle et al. (2015), (3) Chick et al. (2016), (4) Geiger et al. (2012), (5) Khan et al. (2013), (6) Wu et al. (2013), (7) Williams et al. (2016), and (8) Guo et al. (2015). More details on the datasets are given in Table S1. (B) The shift in Pearson correlation values (x axis) for complex-associated proteins (dark red) relative to the background correlation values (gray) is illustrated for the top 6 datasets derived from (A) as density graphs (AUC >0.7). The p value (indicated as *P*) indicates how significant the correlation shift is (one-tailed Mann-Whitney U test). See also Figures S2 and S6 and Table S2.

We assessed the power of each dataset for discovering functional modules by calculating the level of observed co-abundance for known protein-protein interactions utilizing the STRING v10.5 resource (Szklarczyk et al., 2017) and comparing the results to random associations (Figure S1). As expected, throughout all datasets, we recovered pairs of proteins connected by high-confidence interactions (STRING combined score >700) to be more co-abundant across conditions or individuals than protein pairs with no known interactions (see STAR Methods). To further dissect the functional relevance of co-abundant protein sets, we added contextual information about chromosomal location, housekeeping roles (Eisenberg and Levanon, 2013), cellular compartment (Human Protein Atlas) (Uhlén et al., 2015), essentiality (Wang et al., 2015), pathways (Reactome), and protein complexes (Figure 2A; Table S2). The latter were derived from a manually curated list of 279 largely non-overlapping protein complexes as defined by Ori et al. (2016). For each category of contextual information, we assessed using receiver operating characteristic (ROC) curves whether the respective dataset reliably recovered known functional entities, based solely on the co-abundance or co-expression metric. This approach implicitly allows a dual assessment of (1) the overall power of each dataset based on the amount of co-abundance, and (2) an unbiased assessment of the type of functional module yielding the highest level of co-abundance across datasets. With regard to (1), we observed datasets derived from tissue samples to be noisier when compared to cell lines, probably due to the mixture of different cell types within a tissue. Proteomics datasets tended to more clearly recover functional modules as compared to RNA sequencing (RNA-seq) or ribosome profiling datasets (average p = 3.73 × 10^−5^, one-sided Mann-Whitney U test), in line with previous work suggesting an important role of post-translational mechanisms in shaping protein complex abundance and stoichiometry across cell types (Ori et al., 2016).

We observed a consistently high level of co-abundance of members of the same protein complex in proteomics datasets (average p = 8.54 × 10^−4^, one-sided Mann-Whitney U test, Figure 2A). Proteins in other modules, such as pathways, organelles, the housekeeping proteome, etc. showed less coherence on average (average area under curve [AUC] <0.55) (Figure 2A). The shifts toward higher co-abundance were especially apparent in the TCGA proteomics panels, the healthy human individuals (Battle et al., 2015) and the DO and Founder mouse strains (Chick et al., 2016) (Figure 2B). Recent reports (Gonçalves et al., 2017, Roumeliotis et al., 2017) have demonstrated protein complex attenuation due to copy number variations common to cancer and aneuploidy (Liu et al., 2017). Strikingly, the abundance shift for healthy individuals was in some cases even more pronounced than for the cancer-derived datasets, confirming that co-regulation of protein complex members beyond transcription is indeed an inherent cellular mechanism that is preserved across individuals independently of genetic alterations (Figure 2B). Thus, our analysis points toward consistent recovery of protein complexes as the most co-abundant entities within proteomics datasets. To validate the concept externally, but also test for its generality, we applied an analogous work flow to various published datasets on different yeast strains subjected to several environmental conditions. In these datasets, we observed strong and significant co-abundance of complex members across strains (Figure S6A).

### Protein Complexes Vary in Their Stoichiometry across Individuals

**Figure S2:**
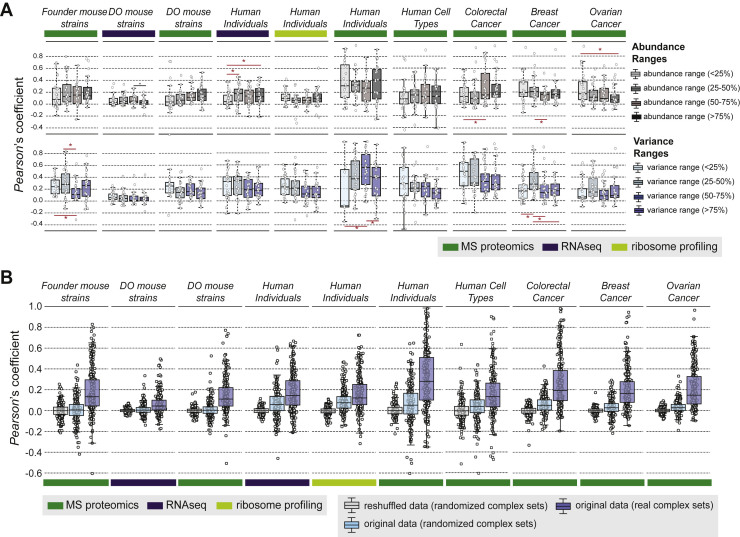
Technical Bias in Abundance Assessment and Complex Correlations, Related to Figure 2 (A) For the datasets as indicated by their respective labeling, it is assessed whether the complex median correlation (Pearson’s *r*) is biased by the abundance of the respective complex (first row, gray shading) or by the complex variance (second row, blue shading). For comparability, abundances and variances are rank-sorted and further split into 25%-bins; the median correlation is then monitored in each bin as a boxplot. The boxplots indicate the median (central line), the IQR (box), and 1.5 times the IQR (whiskers). While they were significant differences between some bins (t test, < 0.1 (^∗^), < 0.05 (^∗∗^), < 0.01(^∗∗∗^)), no general trend could be observed and also those significances could not be recovered consistently across datasets. (B) For the same datasets as above, median correlation values (Pearson’s *r*) were monitored for randomly assembled complexes (decoy complexes) from permuted data (reshuffled data) (gray, first boxplot), decoy complexes from original data (light-blue), and real complex sets from original data (purple). Boxplots indicate the respective median (central line), the IQR (box), and 1.5 times the IQR (whiskers).

**Figure 3:**
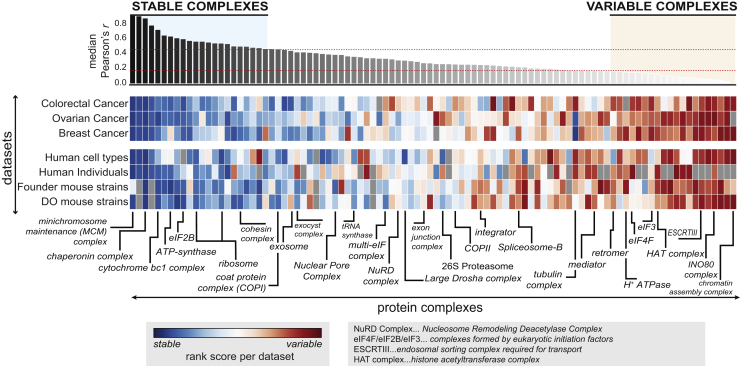
Co-variation Landscape of Protein Complexes in Different Proteomics Datasets Manually curated complexes are shown on the x axis and are sorted according to the median co-abundance observed for complex members in different datasets considered (y axis). Of the original datasets analyzed in Figure 2, the top 6 proteomic datasets that were the best at recovering known functional modules and “human cell types” (used as reference; Geiger et al., 2012) are shown. In each dataset, the median co-abundance of each complex is calculated and ranked. The heatmap illustrates those ranked values, ranging from variable complexes (red) to stable complexes (blue). The top stable and top variable complexes are defined as the respective quantiles (top 25%, variable complexes; bottom 25%, stable complexes). Selected protein complexes are indicated, in some cases using complex name abbreviations as explained in the respective legend. See also Figure S3 and Table S3.

**Figure S3:**
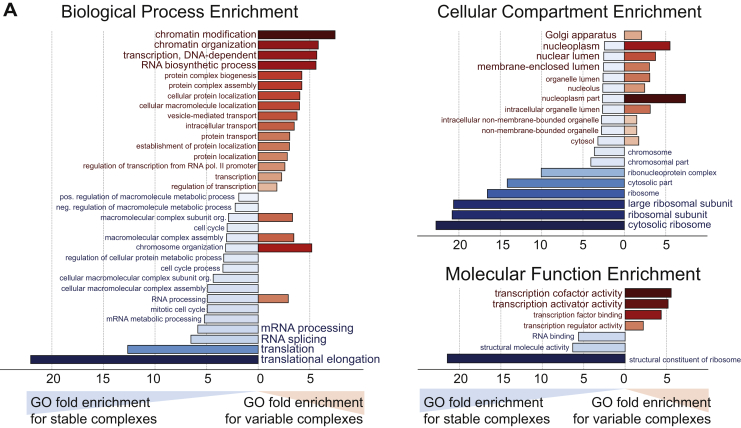
GO-Enrichment Analysis of Stable and Variable Protein Complexes, Related to Figure 3 (A) GO-enrichment analysis in 3 categories (Biological Processes, Cellular Compartment and Molecular Function) delineating the functional differences between stable and variable complexes as recovered from Figure 3. The x axis represents fold-enrichment for stable complexes to the left (blue), whereas to the right fold-enrichments are shown for variable complexes (red). Color opacity correlates with the fold-changes. Only GO-terms with an FDR < 1% (Benjamini-Hochberg) are shown.

**Figure 4:**
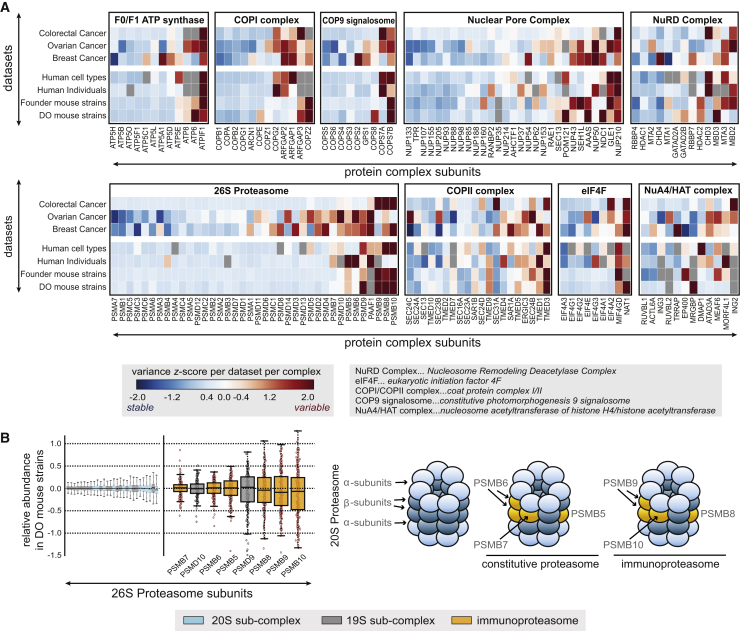
Dissection of Protein Complexes in Stable and Variable Components Reveals Consistent Architecture across Datasets (A) Illustration of stable and variable components in a number of exemplary complexes (x axis: protein complex subunits, y axis: datasets). The heatmaps display *Z* scores, which were calculated based on protein variances following complex normalization (variable component [red]: *Z* score >1.5; stable component [blue]: *Z* score < −1.5). If a given protein was not detected in a dataset, the field is left out gray in the heatmap. Abbreviations of complex names are explained in the respective legend. (B) Members of the immuno-proteasome make up the most variable part of the proteasome complex across individuals, in the displayed example across the diversity outbred (DO) mouse strains. The boxplots (left) display the complex-normalized protein abundances, with the color code highlighting the respective structural entities of the 26S proteasome as indicated in the right-hand cartoon (blue, 20S; gray, 19S; orange, immunoproteasome). Boxplots represent the data median, the interquartile range (IQR, box), and 1.5 times the IQR (whiskers). See also Table S3.

Given the strong signal of variation in complex abundance across individuals in comparison to other functional entities, we focused on a detailed analysis of protein complexes and their stoichiometry in order to identify genetic and environmental factors associated with it. For this purpose, we examined only the proteomics datasets that were yielding the highest recovery of known functional entities due to co-abundance, namely all TCGA cancer datasets, and the datasets on human individuals (Battle et al., 2015) as well as Founder and DO mouse strains (Chick et al., 2016) (p = 2.71 × 10^−6^, one-sided Mann-Whitney U test) (Figure 2B). Using median co-abundance of members in a complex as a proxy for stoichiometric variability across individuals and controlling for a number of technical biases and possible artifacts (Figures S2A and S2B), we recovered a protein complex variability landscape (Figure 3) that is highly consistent across the different proteomics datasets (average Spearman’s rho *=* 0.585; p = 2.88 × 10^−23^, two-sided t test compared to random permutations). We ranked protein complexes according to their level of co-abundance across individuals and identified a subset that is rigorously maintained in stoichiometry (Figure 3). Of 96 well-defined protein complexes with at least 5 protein members, 21 exhibited a tight co-regulation of all its subunits across diseased, as well as healthy individuals (median Pearson’s *r* per complex > 0.46 [75^th^ percentile]). They included the mini-chromosome maintenance (MCM) complex, complexes associated with the translational apparatus (ribosome, chaperonin complex, elongation factor eIF2F) and mitochondrial complexes within the electron transport chain, such as the F0/F1 ATP synthase, cytochrome *bc1* complex, and the cytochrome *c*-oxidase. Variable complexes (median Pearson’s *r* < 0.17 [25^th^ percentile]), on the other hand, were enriched in chromatin-associated processes (Figure S3, false discovery rate [FDR] = 4.68 × 10^−34^, Fisher’s exact test) such as the RNA polymerase, the mediator complex, the BAF complex, etc. The range of complexes in between the two extremes represented instances where both co-regulated parts of complex, as well as more variable members are present, such as in the COPI/COPII, the nuclear pore complex, and the 26S proteasome. We observed a high consistency between datasets after the dissection of modules into stable and variable sub-parts (average Pearson’s *r* = 0.21, p = 9.75 × 10^−15^, two-sided t test compared to random permutations). Variable components, if identified at p value <0.1, made up 2%–20% of the overall structure of the complexes (STAR Methods; Table S3). We found multiple instances of variable complex components consistent with known biology (Table S3). For example, the F1/F0 ATP-synthase inhibitor ATPIF1 was consistently recovered as variable relative to the rest of the ATP synthase complex (Figure 4A, FDR-corrected p = 2.10 × 10^−7^, one-sided t test). ATPIF1 is known to be the master regulator of the F0/F1 ATP-synthase (García-Bermúdez and Cuezva, 2016). Its binding to the complex impedes the hydrolase activity of the ATP-synthase, effectively shutting down its activity to prevent excess wasting of ATP. The observed high variability of ATPIF1 across individuals could thus be explained by the variable energy requirements of the cell (Sánchez-Aragó et al., 2013). Variable members of the nuclear pore complex (NPC) are peripherally associated to the core scaffold, such as e.g., all three transmembrane nucleoporins; varying expression levels of the latter have been implied in differentiation and malignant transformation (NUP210, NDC1, and POM121; FDR-corrected p values p = 8.01 × 10^−11^, p = 0.0643, and p = 0.0644, one-sided t test) (Raices and D’Angelo, 2012). Further variable members of the NPC were found to be ALADIN (AAAS) (p = 0.12, one-sided t test), which potentially binds to transmembrane nucleoporins and has been linked to genetic disease, as well as NUP50 (FDR-corrected p = 0.022, one-sided t test), a subunit involved in active nuclear import (Beck and Hurt, 2017). We also found that paralogous subunits are often variable, such as the ARFGAP-subunits of the COPI complex (average p = 0.043, one-sided t test), the MBD2/3-paralogs involved in the NuRD complex (average p = 0.083, one-sided t test), as well as COPS7A/COPS7B in the COP9 signalosome (average p = 0.010, one-sided t test). This observation is in line with the report from Ori et al. (2016), where paralog switching between different cell types has been described as a major driver for complex re-arrangements.

Another example is the 26S proteasome, in which specific subunits are highly variable across healthy individuals, mouse strains, and cancer patients (Figure 4B). The 20S components PSMB8/PSMB9/PSMB10 of the immunoproteasome, a specific sub-complex of the proteasome involved in immune-regulatory response (McCarthy and Weinberg, 2015), fluctuate the most in their relative abundance within the complex (Figure 4B, average p = 2.82 × 10^−4^, one-sided t test). This sub-complex is known to be replaced by PSMB5, PSMB6, and PSMB7 depending on the cellular context, and these components also vary strongly across individuals (Figure 4B, average p = 0.13, one-sided t test), in multiple different datasets, implying that context-dependent fine-tuning of proteasome activity across individuals is surprisingly prevalent.

**Figure 5:**
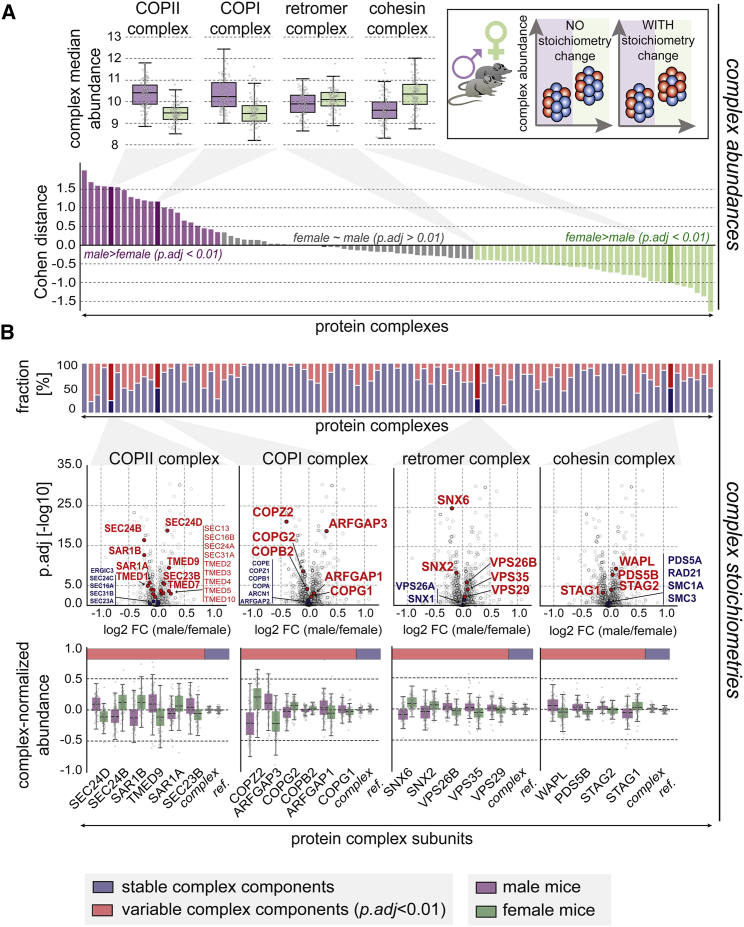
Sex-Specific Regulation of Complex Abundance and Stoichiometry (A) Delineation of differential abundance of protein complexes between male (purple) and female (green) DO mice. The effect sizes (Cohen distance) is shown across all 96 complexes with colors corresponding to significant effects (FDR-corrected p value <0.01 [denoted as *p.adj*], two-tailed t test). Complex median abundances for selected examples are highlighted in the boxplots above the Cohen-distance barplots. These boxplots represent the data median, the IQR (box), and 1.5 times the IQR (whiskers). The inset (upper right) illustrates the concepts of abundance variation and stoichiometry of protein modules. (B) For each complex, the fraction of stable components (blue, not changing in stoichiometry between male and female mice) and differential stoichiometric hits (red) are shown. The volcano plots beneath illustrate the underlying data with log2-fold changes (denoted as *FC*, male/female, x axis) and the adjusted p value on the y axis. Complex-normalized abundances are shown below, highlighting male and female stoichiometry within complexes for variable complex members (red). The stable components (blue) are summarized as *complex ref*. See also Figure S4 and Table S4.

We conclude that proteotype data could be used to predict multi-functionality of sub-complexes or complex components. As individuals vary both in complex abundance and in their stoichiometry, we tried to identify potential genetic and environmental determinants that can at least partially explain this proteotype feature. We primarily leveraged the well-defined meta-data available for DO (diversity outbred) mice strains, namely their sex and their diet, with half of the animals fed with rodent chow and the other with high-fat diet (Chick et al., 2016). We captured two different readouts, namely the variability in (1) complex abundances, and (2) complex stoichiometries (inset in Figure 5A, upper right).

### Sex- and Diet-Specific Protein Complex Abundances

**Figure S4:**
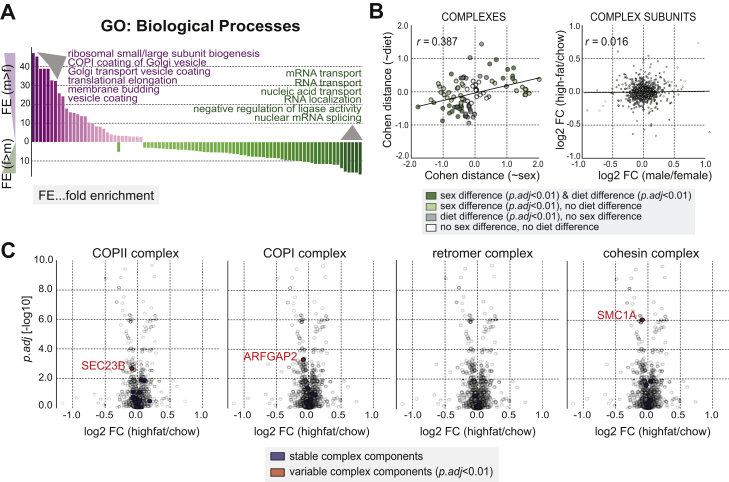
Module Abundance and Stoichiometry Changes Affect Distinct Functional Processes, Related to Figure 5 (A) GO-enrichment analysis (biological processes, see methods) for complexes that are either more abundant in male (purple) or female (green) mice (FDR < 1%, Fisher’s exact test). The x axis shows the individual GO-biological processes that were found to be enriched in male or female mice; the y axis shows the fold-enrichment (*FE*) for each of the processes (upper part: higher enrichment in male versus female; lower part: higher enrichment in female versus male). (B) (*left*) Scatterplot displaying the Cohen distances for sex- (x axis) and diet-differences (y axis) in complex median abundance. (*right*) Stoichiometry changes (LIMMA-derived log2 fold-changes) for male/female differences (x axis) and high-fat/chow differences (y axis) are compared for all complex members. (C) Sex-specific stoichiometry of complexes is not influenced by diet differences. Volcano plots illustrate diet differences in stoichiometry instead of differences due to genetic sex (as shown in Figure 5B).

For complex abundances, differences between male and female mice were evaluated using a standard t test and Cohen distances to yield effect size estimates for each complex (Figure 5A, top). From all 96 considered complexes, 21 complexes showed an overall higher abundance in male mice, while 36 were more abundant in females (FDR-corrected p < 0.01, two-sided t test). Those complexes were enriched in complementary functional processes: Whereas complexes that were more abundant in males were part of the translational process (ribosome, eukaryotic translational factor 2B complex) and specifically protein transport processes involving COPI and COPII, complexes that were more abundant in females, were enriched in mRNA transport and splicing processes (FDR < 0.01, Fisher’s exact test, Figure S4A). This functional complement is indicative of a genetic influence on the abundance of entire complex entities, although we cannot rule out implicit hormonal, life style, or behavioral differences that come along with the different sexes.

The effect of diet on protein complex abundance, on the other hand, was less pronounced; out of 96 considered complexes, 7 complexes had a higher abundance in high-fat diet fed mice and 16 were more abundant in mice exposed to chow (FDR-corrected p < 0.01, two-sided t test). These complexes were mainly enriched in mitochondrial functions and RNA-processing, primarily spliceosome-related sub-complexes.

### Sex- and Diet-Specific Protein Complex Stoichiometries

We next tested if diet and sex influence complex stoichiometry. To this end, a LIMMA-analysis (Ritchie et al., 2015) was performed on complex-normalized abundances for each complex separately (STAR Methods; Table S4) (Ori et al., 2016). Generally, changes in complex abundance did not significantly correlate with the variability in complex stoichiometry (specified as the fraction of subunits affected, FDR < 0.01, Pearson’s *r* = −0.03). For example, the retromer complex only yielded a minimal signal with regard to complex abundance (Figure 5A) but displays a different complex stoichiometry between male and female mice (Figure 5B). In more general terms, a diverse range of functions was variable in complex stoichiometry, including ubiquitin protein ligase activity, mRNA splicing, catabolic processes, and protein transport functions. Within protein transport, in particular, the COPI and COPII complex were largely affected in their relative stoichiometry between male and female mice (FDR-corrected p value of 8.69 × 10^−44^, LIMMA-based t test; Figure 5B). Paralogous components, for example SEC24A/SEC24B/SEC24C/SEC24D—while unaffected between the different diet conditions (Figures S4B and S4C)—contributed to very distinct sex-specific stoichiometry, with SEC24D being consistently more abundant than SEC24B in males and vice versa in females (average FDR-corrected p = 1.40 × 10^−17^, LIMMA-based t test; Figure 5B). SEC24A and SEC24C, on the other hand, had similar complex-relative abundance between male and female mice (average FDR-corrected p = 0.058, LIMMA-based t test). Such sex-specific stoichiometric differences between individuals could indeed have severe functional implications, such as the efficiency or specificity of receptor transport, which has been shown to be affected by the absence and concentration of the specified paralogs (Scharaw et al., 2016). More specifically, the transport of newly synthesized epidermal growth factor receptors (EGFR) from the endoplasmic reticulum (ER) to the plasma membrane coincides with the upregulation of the isoforms SEC24B and SEC24D (Scharaw et al., 2016). We incidentally recovered a significantly high correlation of EGFR with one of those isoforms, SEC24D (Pearson’s *r* = 0.687, p = 1.03 × 10^−30^, two-sided t test compared to random permutations). The functional consequences of the observed changes in complex stoichiometry and their propagation to other cellular processes within an individual remain to be explored. Similar effects were also observed within pathways where the stoichiometry of their members is functionally relevant, such as kinase signaling (FDR-corrected p value of 2.18 × 10^−31^, LIMMA-based t test) (Table S4).

### Effect-Size Estimates of Sex and Diet on Protein Complexes and Functional Modules

**Figure 6:**
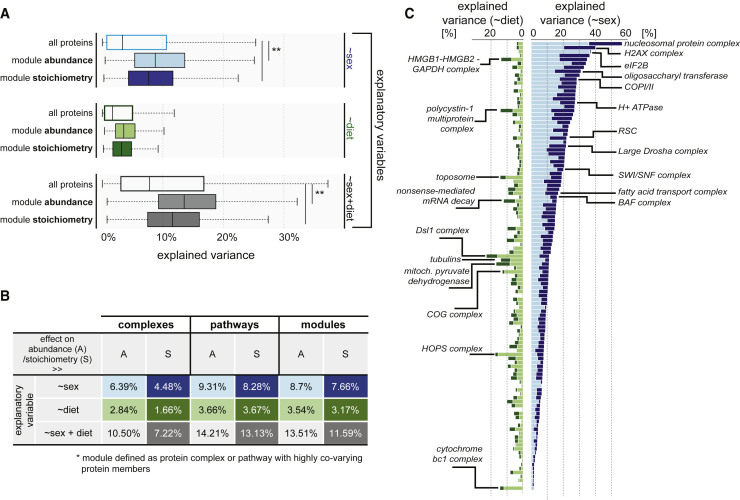
Effects of Sex and Diet on Protein Variation as well as Variation in Module Abundance and Stoichiometry (A) Distribution of the overall effect of sex, diet, and the cumulative effect (sex + diet) on protein abundance variation (all proteins), as well as abundance and stoichiometry variation of modules, including protein complexes and pathways with highly co-varying protein members (see STAR Methods). The lighter colors correspond to effects on abundances, whereas darker colors correspond to effects on module stoichiometry. The boxplots indicate the median (central line), the IQR (box), and 1.5 times the IQR (whiskers). (B) Table displaying median effect sizes of respective variables on complexes only, pathways only, and modules, comprising both complexes and pathways. (C) Distribution of sex- and diet-dependent effect sizes on all complexes (with ≥5 protein members), with lighter colors illustrating effects on abundances and darker colors effects on stoichiometry (see legend from A). Selected complexes showing high degree of variability explained by either sex or diet are highlighted. See also Figures S5 and S6 and Tables S5 and S6.

To quantify the impact of both sex and diet on the overall proteotype, we estimated the effect sizes of those two factors given the observed variation (Figure 6A). On average, less than 5% of the variation of individual protein abundances—regardless of their functional and structural context—was explained by sex differences and even less so by diet differences (∼2%). Some proteins, however, were strongly influenced by the sex of the animal, i.e., SULT2A1 (63.65%, p = 5.88 × 10^−8^, permutation test) and PAPSS2 (64.82%, p = 3.20 × 10^−8^, permutation test), which are crucial for sulfation of the androgen precursor (Oostdijk et al., 2015).

**Figure S5:**
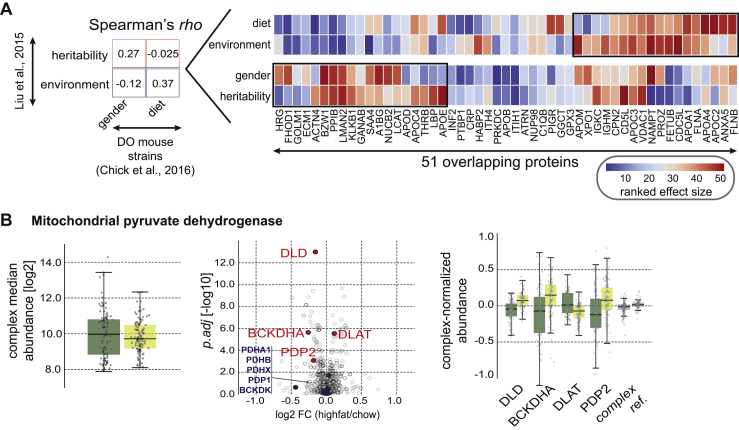
Comparison of Effect-Size Landscape and Stoichiometric Changes in the Mitochondrial Pyruvate Dehydrogenase Due to Differential Diet, Related to Figure 6 (A) (*left*) Summary of Spearman’s correlation values between effect sizes on proteins derived from Liu et al. (2014), and effect sizes calculated for proteome of DO mouse strains. (*right*) The heatmap displays the ranked effect sizes for 51 proteins that were quantified in both Liu et al. (2015), and DO mouse strains (red: stronger effect; blue: lower effect). The factors affecting proteins are listed on the left side of the heatmap. (B) Diet-specific stoichiometry of the mitochondrial pyruvate dehydrogenase: (*left*) the overall complex median abundance is not affected, (*center*) volcano plot highlighting the complex-specific fold-changes of particular subunits of the complex, (*right*) complex-normalized abundances with enhanced differentially expressed proteins (high-fat = dark green, chow = light green). All presented boxplots indicate the respective median (central line), the IQR (box), and 1.5 times the IQR (whiskers).

To see whether the effect of the animal’s sex on protein abundances is reflective of the underlying genetic factors, we compared the obtained effect sizes directly with the results from Liu et al. (2015), a study on identical twins that addressed to what extent the variation of 342 human plasma proteins can be explained by genetic factors, environment, and age. We found that 37% of the environmental effect on the human plasma proteome could be recovered in the DO mice as diet-dependent (Spearman’s rho = 0.37, p = 4.85 × 10^−3^, two-sided t test) (Figure S5A). The genetic effect on the human plasma proteome and the impact of the animal’s sex on protein variation correlated positively as well (Spearman’s rho = 0.27, p = 3.06 × 10^−2^, two-sided t test).

We hypothesized that effect sizes might be higher for functional modules and estimated to what extent the variation of such co-abundant modules, including both protein complexes and highly co-abundant pathways, is influenced by each of the two or both. For this, we considered the two above-described metrics, abundance and stoichiometry. On average, sex and diet cumulatively explained 13.51% of the abundance variation in functional modules (Figure 6A), with sex explaining on average 8.7% and diet only 3.54%. Some pathways, such as the complement pathway, were strongly affected by these co-variates: for example, around 38.8% of the variation in the abundance of the complement pathway was due to the animal’s sex (p = 4.95 × 10^−7^, permutation test). Other pathways, including androgen and glucocorticoid biosynthesis, had an expectedly large fraction of their abundance variation explained by sex differences (38.36% [p = 6.80 × 10^−7^] and 32% [p = 6.66 × 10^−5^]; permutation test) (Table S5). The effects of diet on pathway abundances, on the other hand, were primarily apparent for metabolic pathways, such as the urea cycle (28.69%, p = 6.95 × 10^−4^, permutation test) and cholesterol biosynthesis (19.04%, p = 0.068, permutation test). For protein complexes, we could recover a similar span of effect sizes: on average, 10.50% of the abundance variation of complexes could be ascribed to the cumulative effect of the sex and diet of the animals. Some complexes, however, were affected by neither (<1%), whereas other complexes had more than 30% of their abundance variation explained by these co-variates (Figure 6C). For example, the sex of the mice explains up to 35% of the variance in the abundance of the eIF2B multi-subunit complex (36.62%, p = 1.75 × 10^−10^, permutation test) and the nucleosome protein complex (36%, p = 2.43 × 10^−10^, permutation test), as deduced from co-variate analysis controlling for diet. On the other hand, the effect sizes of diet on protein complex median abundances reached up to only 15% and were generally restricted to another set of complexes, such as the Dsl1p complex (15.88%, p = 0.042, permutation test), the HOPS complex (15.63%, p = 0.048, permutation test) and mitochondrial complexes, e.g., the cytochrome *bc1* complex (11.94%) (Figure 6C).

Variation in modular stoichiometry, comprising both complexes and pathways, was on average less influenced by either factor (3.17%–7.66%) (Figure 6B; Table S5). However, some pathways and complexes were considerably more affected in their stoichiometry by diet than by sex (Figure 6C; Table S5). One striking example is the mitochondrial pyruvate dehydrogenase complex: the median abundance of the complex was the same regardless of the two explaining variables (Figure S5B); the stoichiometry was not substantially affected by the sex of the animals (4.73%, p = 0.20, permutation test), yet diet impacted it significantly (effect size of 8.73%, p = 1.847 × 10^−3^, permutation test). Specifically, the subunits DLD and BCKDHA were found to be on average higher in abundance under chow diet conditions as compared to high-fat diet conditions (Figure S5B).

The general lack of correlation between sex and diet-specific stoichiometry changes (Pearson’s *r* = 0.016), underscores the functional complementarity of genetic and environmental factors (Figure S4B) and implies a possibility to revert changes caused by environmental effects.

**Figure S6:**
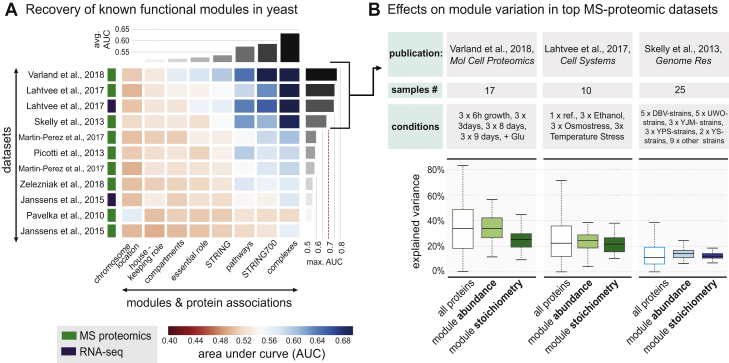
Genetic and Environmental Effects on Functional Module Variation in Yeast, Related to Figures 2 and 6 (A) Recovery of known functional modules by means of receiver operating characteristic (ROC)-analysis. Each cell of the matrix displays the AUC (area under curve) value for a given module (x axis) in the given dataset (y axis). Modules of protein associations are ordered according to respective average AUCs. The type of data is indicated by the colored boxes next to the dataset. The datasets have been extracted from the following publications: (1) Varland et al. (2018), (2+3) Lahtvee et al. (2017), (4) Skelly et al. (2013), (5+6) Martin-Perez and Villén, 2017, (7) Picotti et al. (2013), (8) Zelezniak et al. (2018), (9+10) Janssens et al. (2015), and (11) Pavelka et al. (2010). (B) Table displaying details for top proteomics yeast publications, sample numbers and conditions. On the left-hand side the distribution of the overall effect of environment (green) and genetics (blue) on protein abundance variation (all proteins) is shown, as well as abundance and stoichiometry variation of modules, including protein complexes and pathways with highly co-varying protein members. The lighter colors correspond to effects on abundances whereas darker colors correspond to effects on module stoichiometry. The boxplots indicate the respective median (central line), the IQR (box), and 1.5 times the IQR (whiskers).

Although not directly comparable, our analogous analysis of yeast strains (Figure S6B) supports our findings in mammals. The more extreme environmental conditions tested in yeast (glucose starvation and ethanol, osmotic, and/or temperature stress) impacted functional modules much more dramatically, explaining on average 25% of the observed module variation. The impact of environmental factors was substantially higher than the one of genetic diversity between yeast strains (on average 13%, see Figure S6B), which points to an even larger source of molecular markers for environmental impact on individuals.

## Discussion

Here, we provide a systematic analysis of unrelated MS-shotgun proteomic datasets, revealing widespread variation of abundance and stoichiometry of pathways and complexes. Leveraging the modular architecture of the proteome, we show that some complexes have a stable composition throughout different proteomic datasets, whereas other complexes are subject to considerable variation across cell types as well as individuals. We illustrate that this observed variability is partly due to the presence of specific variable sub-parts of complexes that are adapted for moonlighting purposes or fine-tuned to cellular conditions. The variability of the immunoproteasome, for example, coincides with the abundance levels of components involved in the immune-regulatory response. Consistently stable complexes, on the other hand, are primarily subject to structural constraints and rigorous stoichiometric control. So far, both degradation of unbound subunits (McShane et al., 2016, Ryan et al., 2017), as well as regulatory mechanisms at the RNA level (Wu et al., 2013) have been suggested as major drivers of stoichiometric robustness in complexes. Such mechanisms are not restricted to protein complexes. Specific pathways, such as the complement pathway, display a very high and consistent co-variation of all its protein members involved. Proteins in central cellular pathways such as the citric acid cycle are also stable in their relative stoichiometry. In the future, such pathways might be further interrogated to understand which parts are subject to careful stoichiometric maintenance and what variable members contribute to differential proteotypes.

While it has been previously shown that slight architectural changes of complexes and pathways occur between different cell types due to respective cellular morphologies and gene expression programs (Ori et al., 2013), our analysis reveals that such changes even become apparent across individuals within the same cell type. This raises the questions to what extent complex and pathway stoichiometry is determined by cell differentiation programs and which other factors contribute to or confine structural arrangements of functional protein modules. We focus on two factors, the genetic sex of the individual and two distinct diets, to quantify examples of both genetic and environmental factors as potential sources of variation of module abundance and stoichiometry. The effect sizes on variation in module abundances were usually larger, with an average of >5%, than in module stoichiometry (average <5%), which was expected due to the rigor in module architecture. The impact of an individual’s sex on the proteotype are currently largely centered around well-known mechanisms such as dosage compensation and differential expression of proteins due to their X/Y chromosome location (Wu et al., 2013, Chick et al., 2016). Our analysis identifies a considerable number of sex-specific variations in complexes and pathways that go beyond the location of sex-specific chromosomes. At this point, it remains unclear, however, whether the given stoichiometry has a truly genetic cause that can be traced back to X- and Y-associated gene expression, or whether it emerges via indirect environmental effects, such as the influence of hormones or life styles. The observed effect size of diet was overall smaller; however, a few protein complexes are predominantly affected, very often in metabolic context. Our findings suggest that in these specific cases changes of diet might be exploited to counteract other factors in order to favorably adjust an individual’s proteotype.

Using yeast strains exposed to various, more drastic, environmental conditions, we could indeed demonstrate that the environmental imprint on the proteome appears to be much higher, explaining as much as 25% of the observed variation in protein complex composition and stoichiometry, albeit in the context of a high genetic diversity, which explained 13% of the respective variation.

Although somewhat limited in environmental condition on mammalian individuals, our analysis provides a stepping stone in defining the underlying determinants of variation in the proteome, which could arguably be exploited in future diagnostic and clinical contexts. The proteotype of an individual represents the most direct readout for the functional state of cells, as protein abundance and module stoichiometry are the result of prior integrative processes at the transcriptional, translational, and post-translational level. Establishing whether certain cellular processes caused by disease are due to genetic or environmental differences is key to stratifying patient cohorts and might provide a framework for personalized medicine. Even subtle differences in stoichiometric set-ups of protein modules could have systemic effects on the entire proteome and affect cellular logistics and organelle composition, as illustrated by the observed changes in the nuclear pore and vesicle trafficking systems. By establishing the network of interdependencies between complex and pathway stoichiometry and an individual’s phenotype, the proteotype could indeed be fully leveraged as a functional readout for disease risk assessment in the future.

## STAR★Methods

### Key Resources Table

**Table:** 

REAGENT or RESOURCE	SOURCE	IDENTIFIER
**Deposited Data**		

Proteomics Datasets, RNA-seq and Ribosome Profiling	Web Resource	http://www.bork.embl.de/Docu/proteotype_genetic_environment_impact/download.html

**Software and Algorithms**		

R	The R Project	https://www.R-project.org; RRID: SCR_001905
Bioconductor	Bioconductor	https://www.bioconductor.org; RRID: SCR_006442
Python 2.7	Python Software Foundation. Python Language Reference, version 2.7	https://www.python.org; RRID: SCR_008394
UNIPROT	UniProt Consortium	https://www.uniprot.org:443/; RRID: SCR_002380
Scikit-learn: Machine Learning in Python	scikit-learn: Machine Learning in Python	https://scikit-learn.org/stable/index.html; RRID: SCR_002577
Bioconductor Package: Linear Models for Microarray Data (LIMMA)	Ritchie et al., 2015	https://bioconductor.org/packages/release/bioc/html/limma.html; RRID: SCR_010943
MyGene, Gene Annotation Service	mygene.info	https://pypi.org/project/mygene/
DAVID Bioinformatics Resources 6.8	DAVID, NCI	https://david-d.ncifcrf.gov/; RRID: SCR_001881

**Other**		

Resource website for data analysis	Web Resource	http://www.bork.embl.de/Docu/proteotype_genetic_environment_impact/
Reactome Pathway Database	Reactome	https://reactome.org; RRID: SCR_003485
Complex Database	Ori et al., 2016	http://www.bork.embl.de/Docu/variable_complexes/
The Human Protein Atlas	Uhlén et al., 2015	http://www.proteinatlas.org; RRID: SCR_006710
Saccharomyces Genome Database	SGD community	https://www.yeastgenome.org/; RRID: SCR_004694
STRING database (version 10.5)	Szklarczyk et al., 2017	https://string-db.org/cgi/input.pl; RRID: SCR_005223

### Contact for Reagent and Resource Sharing

Further information and requests for resources should be directed to and will be fulfilled by the Lead Contact, Peer Bork (peer.bork@embl.de).

### Experimental Model and Subject Details

The analyzed data are derived from a number of organisms and cell lines: (i) A549, GAMG, HEK293, HeLa, HepG2, K562, MCF7, RKO, U2OS, LnCap and Jurkat cells (Geiger et al., 2012), (ii) kidney tissue samples (tumor and matched kidney tissues) (Guo et al., 2015), (iii) EBV-transformed lymphoblastoid cell lines (LCLs) derived from 5 human (YRI), 5 chimpanzee individuals, LCLs from 5 rhesus macaque individuals (Khan et al., 2013), (iv) EBV-transformed LCLs derived from humans (YRI, Yoruban people) (Battle et al., 2015), (v) BXD mouse strains on chow diet (CD) and high-fat diet (HFD) (Williams et al., 2016), (vi) Diversity Outbred mice (DO) from Jackson Laboratory (JAX) with 3 weeks of age, exposed to chow diet (CD) or high-fat diet (HFD) (Chick et al., 2016), (vii) LCLs from 95 HapMap individuals (Wu et al., 2013), (viii) tumor samples derived from the TCGA Biospecimen Core Resource (Zhang et al., 2014, Mertins et al., 2016, Roumeliotis et al., 2017). More details on additional experimental features are given in Table S1.

### Method Details

The underlying data and code for each processing step can be found in the following web resource: http://www.bork.embl.de/Docu/proteotype_genetic_environment_impact/.

#### Information Resources and Integration of Data

Protein-protein interactions were obtained from the STRING database (*version* 10.5) (Szklarczyk et al., 2017); interactions were considered to exist if the (STRING) combined score > 0, to be confident if combined score > 0.5 (Figure S1), and high-confidence interactions if combined score > 0.7. The database of complexes was manually compiled and curated from COMPLEAT and CORUM by Ori et al. (2016), and quantified proteins from all published datasets considered for the analysis were mapped accordingly. Pathways were obtained from the Reactome Pathway Database (downloaded in February, 2017, https://reactome.org/download-data/). Cellular locations were extracted from the Human Protein Atlas (downloaded February 2017) (Uhlén et al., 2015) considering protein mappings only if this assignment has been either validated, supported or confirmed by antibody analysis (keyword ‘approved’). Chromosome locations were mapped using the Python package *mygene* (https://pypi.org/pypi/mygene) using the *hg19* GenBank assembly for human and the *mm10* genome assembly for mice, respectively. Finally, essentiality of genes was defined based on the genetic screen performed in the human cell lines KBM7, K562, Jiyoye, and Raji by Wang et al. (2015) (Table S2); genes with a housekeeping role were obtained from the supplementary files of the report by Eisenberg and Levanon (2013). [Step1 in web resource]

#### Large-scale proteomic datasets

For the delineation of protein abundances across individuals, we primarily considered large-scale shotgun proteomics studies on human individuals, cancer patients and mouse strains. For control purposes, we also included the proteomic profiles of 11 human cell lines generated by Geiger et al. (2012). Technical specificities of each dataset (such as sample number or MS-acquisition), as well as the number of quantified proteins, as well as all required module mappings are given in Table S1. For the 60 Yoruban HapMap individuals (Battle et al., 2015) also the respective data from RNA-seq analysis and ribosome profiling was available and therefore included in the analyses. Data on DO (diversity outbred) mouse strains (Chick et al., 2016) were available at the proteomic as well as transcript level. The cancer proteomics datasets were downloaded from the TCGA CPTAC project (Zhang et al., 2014, Zhang et al., 2016, Mertins et al., 2016).

### Quantification and Statistical Analysis

#### Pre-processing steps

For each dataset we checked whether batch effects or possible normalization issues could have arisen in the published processing [Step2 in web resource]. To this end, each sample was tested for normality using the Shapiro-Wilk test, as this is a standard framework to test for any possible deviations from normality; only in datasets from Geiger et al. (2012), and Guo et al. (2015), samples were detected that showed possible batch effects. In this case, we log-transformed and quantile-normalized those to standardize sample distribution. For each dataset we also checked whether any bias could arise for the calculation of protein correlations based on abundance- and variance-distribution (Figure S2).

#### AUROC analysis

For the Receiver Operating Curve (ROC) analysis across different types of modules in different datasets, condition positives were defined based on the databases as outlined above. The lowest number of condition positives occurring is 1.540 (interactions). For *pathways* we excluded interactions within protein complexes (such as the ribosome complex). When considering *chromosome location*, we defined true positive “interactions” to exist between genes encoded on the same chromosome. For the categories *essentiality* and *housekeeping role* true positive interactions were to occur between essential genes and housekeeping genes, respectively. The full set of condition negatives consists of all other pairs of proteins. For computational reasons, we randomly sampled from the full set of condition negatives the same number of respective condition positives to compute ROC curves. The area under the curve (AUC) value was calculated using the trapezoidal rule. We applied Mann-Whitney *U*-statistics, which is directly connected to the AUC metric, to test whether correlation values derived from proteins that are in the same modules, are significantly different from correlation values derived from random proteins that are not part of any module. To make a conservative estimate of the effect size (and *p-value*), we applied the Mann-Whitney *U*-test 1000 times to a randomly sampled selection of 1000 items from the two distributions, respectively, and calculated the mean *p-value*. [Step6 in web resource]

#### Co-abundance of proteins in complexes

As mentioned above, the database of complexes was manually compiled and curated from COMPLEAT and CORUM by Ori et al. (2016), and quantified proteins from all published datasets considered for the analysis were mapped accordingly. A subset of manually curated protein complexes were classified as ‘well-defined’ (Ori et al., 2016). For further analysis only protein complexes with at least 5 quantified members were considered in each dataset, respectively. Co-abundances of proteins were calculated as Pearson correlations between log-transformed protein abundances across individuals. As a control, proteins that were not part of any complex assembly were randomly assigned into artificial complexes and cross-correlated as well (Figure S2B). In addition, the data was also permuted and proteins subunits were again tested for co-abundance using Pearson correlation (Figure S2B). The Mann-Whitney *U*-test was applied to assess significance. [Step4 in web resource]

#### Gene ontology analysis

For all gene ontology (GO) analyses in this study, respective genes were analyzed using DAVID (version 6.8; https://david-d.ncifcrf.gov). The GO domains ‘Biological Process’, ‘Molecular Function’ and ‘Cellular Compartment’ were considered; the background for the GO-analysis was represented by all quantified proteins in a given dataset. Results were filtered according to FDR (Benjamini-Hochberg) of less than 0.01; the fold-changes associated with those significantly enriched GO-terms are shown (Figures S3 and S4A). [Step9 in web resource]

#### Identification of stable and variable complexes

As a general principle, we used the median co-abundance of proteins within a complex as a proxy to differentiate between stable and variable complexes. To compare the extent of complex stability and variability, correlations were ranked within each dataset; finally the median rank of each complex as recovered from each considered dataset was calculated, and complexes were sorted accordingly. The top quantile (25%) of these complexes were considered to be highly stable (Pearson’s *r* > 0.46), whereas the lowest quantile were considered highly variable (Pearson’s *r* < 0.2). To assess the consistency of the complex variability landscape, we calculated the Spearman correlation of the ranked median co-abundance across datasets (as illustrated in Figure 3). As a reference distribution we permuted the dataset 1000 times, and computed Spearman correlation coefficients across datasets each time. In a two-sided t test we then compared the real distribution of correlation values with the ones derived from the random permutations of the dataset. This testing set-up does not presume any directionality in the hypothesis testing (two-sided) and is justified due to the normality of the reference distribution (as confirmed by the Shapiro-Wilk test). [Step7 in web resource]

#### Protein complex stoichiometry analysis

To assess compositional rearrangements of protein complexes as opposed to their overall abundance changes, a module-wise normalization was performed, as previously described (Ori et al., 2013, Ori et al., 2016). Proteins belonging to the same complex were normalized by the respective trimmed mean (or interquartile mean) of the complex subunits across all individuals/samples. In case of proteins involved in multiple complexes, the average value from all the corresponding complexes was taken into account. Given the complex-normalized abundances, the variance of each subunit in a given complex was calculated. To compare these variances between different proteomics datasets and approaches, those variances were converted to *z*-scores per complex (Figure 4). Similarly to testing the consistency between datasets in the above section, we calculated the correlation coefficients (between datasets) for each such a *z*-score matrix. To compile a reference distribution we permuted each matrix and calculated corresponding correlation coefficients 1000 times, which provides a normal distribution. In a two-sided t test we then compared the real distribution of correlation values with the ones derived from the random permutations of the dataset. Protein subunits within a complex were considered ‘stable’ or ‘variable’ in case of the associated *p-value* < 0.05 based on the distribution of *z*-scores (Table S3). To see whether a given protein is consistently ‘variable’ in a complex throughout all given datasets, the distribution of its *z*-scores within the complex and across all the datasets were compared to the *z*-score distribution for all other protein components of the same complex across all datasets (one-sided t test). A one-sided t test accounts for the unidirectionality of our hypothesis and gives conservative results. This procedure was done for all proteins in each complex, and resulting *p-value*s were adjusted using the Benjamini-Hochberg method. [Step8 in web resource]

#### Sex- and diet-specific abundance changes

To assess the differences in abundances of entire complex structures between male/female mice, and mice exposed to high-fat and chow diet, the median abundances of each complex was calculated in each individual/sample (protein subunits were required to be quantified in at least 50% of samples). For each complex it was then assessed via a t test whether median complex abundances in male mice were significantly different from the ones in female mice; the effect size was monitored as the Cohen distance. The applied t test is two-sided as no directionality is implied. *P*-values were further adjusted using the Benjamini-Hochberg procedure (significance α = 0.05), and complex structures were considered significantly different in case of *q*-value < 0.01. [Step10 in web resource]

#### Sex- and diet-specific stoichiometry changes

For internal rearrangements of the complex (stoichiometry), we performed a separate analysis applying the *R*-package LIMMA (Linear Models for Microarray data analysis) (Ritchie et al., 2015) using the complex-normalized protein abundances as input. LIMMA was applied to give a more conservative variance estimate to allow for robust inference on differences compared to ordinary t tests. Analogous to differential expression analysis, proteins showing a difference in their complex-normalized abundance relative to the other complex members were considered differentially expressed or stoichiometrically different between two given conditions. Contrasts were set accordingly to identify differences between male/female mice and high-fat/chow mice, respectively. For each complex, protein complex members were subjected to stoichiometric analysis; log2 fold-changes as well as *p-value*s (moderated t test) were collected. *P*-values were adjusted using the Benjamini-Hochberg procedure across all complexes and proteins. In case of *q*-value < 0.01 the corresponding protein was considered to be stoichiometrically changing in a given complex. The underlying statistical test is denoted as ‘LIMMA-based t test’ throughout the main text. The corresponding fold-changes are highlighted in volcano plots in Figures 5B, and S3C. The analysis was also performed for Reactome pathways, and can be readily applied to any specified protein set/module. To assess which complexes are affected in their stoichiometry as a whole, *q*-values of their individual components were combined using Fisher’s method. Lastly, the resulting combined *p-value*s from all complexes were adjusted using the Benjamini-Hochberg method. [Step11 in web resource]

#### Effect size estimations on proteins and modules

To understand to what extent both protein complex abundance and stoichiometry are affected by either sex or diet, a L2-regularized Multiple Linear Regression (Ridge regression with a regularization parameter of 1) was used, as implemented in the *scikit* Python package (https://scikit-learn.org). We compared models that predict complex abundance or complex stoichiometries, using as predictors: (i) *genetic sex*, (ii) *diet*, and (iii) the combination of *genetic sex* and *diet* together. We assessed the quality of each model by the coefficient of determination (*R*^*2*^). This was done for every module considered (complexes and pathways), for abundance, as well as module-normalized data. For pathways we only considered those that were showing a high co-abundance (FDR-corrected *p-value* < 0.1) as compared to co-abundances derived from a reshuffled dataset.

To estimate prediction performance we used a 10-fold cross-validation scheme. Briefly, we randomly separated the dataset (per complex) into ten groups of equal size, in order to iteratively train a model with nine of them, and to assess the testing performance in the held-out group. For each module the median global *R*^*2*^ is reported. While the *R*^*2*^-score represents a measure of effect size, an additional target-decoy strategy was applied to estimate the significance of those scores. An identical analysis was conducted with a reshuffled dataset per complex, and the corresponding performance metrics were used in a permutation test approach to assign significance to the true ridge regression coefficients. Specifically, we use the latter distribution to calculate an empirical FDR. Throughout the main text, the global *R*^*2*^ performance metric derived for the module or protein is reported as the effect size with its respective FDR-corrected *p-value*. [Step12 in web resource]

#### Yeast dataset analysis based on co-variation

Additionally to the proteomic datasets derived from mammalian organisms, we also analyzed published MS-datasets of yeast proteomes and their corresponding RNA-seq datasets if available. A total of eight independent publications were considered: (i) Martin-Perez and Villén (2017), (ii) Skelly et al. (2013), (iii) Lahtvee et al. (2017), (iv) Picotti et al. (2013), (v) Pavelka et al. (2010), (vi) Varland et al. (2018), (vii) Zelezniak et al. (2018), (viii) Janssens et al. (2015). 11 datasets derived from these publications (Table S6) were quantile-normalized and filtered according to their potential to recover known protein-protein interactions based on co-variation (Figure S6A; see above section “AUROC analysis”). [Step19 in web resource]

#### Estimation of effect sizes on modules in yeast

This analysis was performed with three yeast proteomic datasets that showed a reliable recovery of known protein-protein interactions (AUC > 0.7), namely (i) Varland et al. (2018), (ii) Lahtvee et al. (2017), and (iii) Skelly et al. (2013). In the datasets (i) and (ii), yeast cells were exposed to different environmental conditions (i.e., osmotic/temperature/ethanol/nutritional stress); dataset (iii) compared genetically diverse yeast strains. To estimate the extent of variation of both protein complex abundance stoichiometry due to these environmental and genetic condition, we used a similar framework as described in the section above (“*Effect size estimations of sex- and diet on proteins and modules”*). Samples for dataset (i) and (ii) were respectively grouped into the environmental conditions, whereas samples for dataset (iii) were grouped into related sets of yeast strains (according to source and collection). For each dataset these categorizations were then tested as predictors for complex abundance and stoichiometry. For each complex, the quality of each model was assessed by the coefficient of determination (*R*^*2*^) in a 10-fold cross-validation scheme, as described above. [Step20 in web resource]

### Data and Software Availability

All scripts for analyzing data and generating figures are available at http://www.bork.embl.de/Docu/proteotype_genetic_environment_impact/. The web resource allows interrogating every step of the computational analysis, with corresponding in- and output data.

## Supporting Citations

The following references appear in the Supplemental Information: Janssens et al. (2015); Lahtvee et al. (2017); Martin-Perez and Villén (2017); Pavelka et al. (2010); Skelly et al. (2013); Varland et al. (2018); Zelezniak et al. (2018).

## Supplemental Information

Table S1. Overview of Used Datasets Including Technical Details and Sample Size, Related to Figure 1

Table S2. Underlying Data for Delineating Module Recovery from Used Datasets, Related to Figure 2

Table S3. Stable and Variable Protein Complex Members for Each Complex, Related to Figures 3 and 4

Table S4. Stoichiometry Variations in Each Dataset for Each Complex, Related to Figure 5

Table S5. Effect Sizes of Sex and Diet and Combined on Proteins and Modules, Related to Figure 6

Table S6. Overview on Yeast Proteomic Datasets and Effect Sizes of Genetic and Environmental Factors on Proteins and Modules, Related to Figures 6 and S6
